# Antisecretory Effect of Hydrogen Sulfide on Gastric Acid Secretion and the Involvement of Nitric Oxide

**DOI:** 10.1155/2014/480921

**Published:** 2014-02-24

**Authors:** Seyyed Ali Mard, Hasan Askari, Niloofar Neisi, Ali Veisi

**Affiliations:** ^1^Research Institute for Infectious Diseases of Digestive System, Physiology Research Center (PRC) and Department of Physiology, The School of Medicine, Ahvaz Jundishapur University of Medical Sciences, Ahvaz 61357 15794, Iran; ^2^Department of Physiology, The School of Medicine, Tehran University of Medical Sciences, Tehran, Iran; ^3^Research Institute for Infectious Diseases of Digestive System, The School of Medicine, Ahvaz Jundishapur University of Medical Sciences, Ahvaz 61357 15794, Iran; ^4^Research Institute for Infectious Diseases of Digestive System and Department of Virology, The School of Medicine, Ahvaz Jundishapur University of Medical Sciences, Ahvaz 61357 15794, Iran

## Abstract

The present study was designed to investigate the effect of H_2_S on distention-induced gastric acid secretion. Fifty-two rats were randomly assigned to seven experimental groups. The gastric acid secretion was stimulated by gastric distention. Two groups of rats received L-cysteine or saline for 5 days before stimulation of the gastric acid secretion. Two groups of animals also received NaHS or saline just prior to stimulation of the gastric acid secretion. The effect of L-NAME and propargylglycine was also investigated. The mucosal levels of the gene expression of cyclooxygenase-2 (COX-2), endothelial nitric oxide synthase (eNOS), and H^+^/K^+^-ATPase **α**-subunit were quantified by qPCR and luminal concentrations of NO were determined. NaHS and L-cysteine decreased the gastric acid output in response to distention. The mRNA expression of H^+^/K^+^-ATPase **α**-subunit decreased by NaHS and L-cysteine as compared with the control group while gene expression of eNOS and COX-2 was upregulated. The inhibitory effect of NaHS on distention-induced gastric acid secretion was mitigated by pretreatment of L-NAME. These findings suggest the involvement of NO in mediating the antisecretory effect of H_2_S.

## 1. Introduction

Hydrogen sulfide (H_2_S) has been shown to act as a new gaseous transmitter in mammalian tissues [1, 2]. Three key enzymes, cystathionine-*γ*-lyase (CSE), cystathionine-*β*-synthase (CBS), and 3-mercaptopyruvate sulfurtransferase (3MST) along with cysteine aminotransferase, are involved in the natural production of H_2_S in the body [3]. A growing body of literatures have documented several physiological roles for H_2_S including vasodilation, neuromodulation, and smooth muscle relaxation [4–6]. Additionally, exogenous H_2_S has been shown to protect the gastric mucosa on different model of experimentally induced gastric ulcer in rats [7–9].

H_2_S has been shown to increase the release of NO from vascular endothelium [10]. Moreover, it has been reported that NaHS, a H_2_S donor, induces the duodenal release of NO in rat [11]. Since NO has been shown to inhibit gastric acid secretion in rabbit, human and rat [12–14], the present study aimed to elucidate the possible mechanism of the inhibitory effect of NaHS, a H_2_S donor, on gastric acid secretion in rats and to determine whether this effect is related to nitric oxide.

## 2. Materials and Methods

### 2.1. Animals

Male Wistar rats (170–220 g) were supplied from the animal house of Ahvaz Jundishapur University of Medical Sciences. Animals were fed on conventional diets and tap water. They were maintained under standard conditions of humidity, temperature (22 ± 2°C), and light/dark cycle (12 h : 12 h). All experiments were carried out in accordance with ethics committee of Ahvaz Jundishapur University of Medical Sciences (number RDC-9102).

### 2.2. Animal Grouping and Surgical Procedures

Animals were anesthetized with a mixture of ketamine and xylazine (60 + 15 mg/kg, i.p.). Depth of anesthesia was monitored throughout the experiment by the pedal withdrawal (toe pinch) reflex every 30–45 min. If the reflex was observed, a supplemental dose of anesthetics was administered to maintain adequate anesthesia. Animal body temperature was controlled with a rectal thermometer and maintained at 37 ± 0.5°C by using a homeothermic blanket control system (Harvard, Edenbridge, UK). After a midline laparotomy, both the stomach and the duodenum were exposed. A polyethylene catheter (3 mm, O.D.) was inserted into the stomach through the duodenum and held in place by a ligature around the pylorus. At the beginning of each experiment, the lumen of the stomach was gently rinsed with isotonic saline (pH 7, 37°C) until gastric washout was clear. In the first set of experiments, forty rats were randomly divided into 5 groups (8 in each). They were control (C), NaHS-, L-NAME + NaHS-, PAG-, and PAG + SNP- (sodium nitroprusside-) treated rats. After determination of the basal acid output, the gastric acid secretion was stimulated by distention (1.5 mL/100 g of body weight by normal saline, pH 7 and 37°C for 90 min) in all groups. In a preliminary study we have shown that NaHS at 20, 40, and 80 *μ*g/kg decreases the gastric acid output in a dose-dependent manner [15]. Therefore, the maximal affected dose of NaHS was used in the present work. To evaluate the effect of H_2_S on distention-induced gastric acid secretion, NaHS-treated rats received an intravenous injection of H_2_S donor, NaHS (80 *μ*g/kg), via a tail vein just prior to stimulation of the gastric acid secretion by distention. To determine the possible role of NO in mediating the inhibitory effect of H_2_S on distention-induced gastric acid secretion, one group of animals, L-NAME + NaHS-treated rats, received N^G^-nitro-L-arginine methyl ester, L-NAME (10 mg/kg, i.v.), 5 min before the administration of NaHS (80 *μ*g/kg, i.v.) [11]. To evaluate the effect of endogenous H_2_S on distention-induced gastric acid secretion, animals in PAG-treated group received cystathionine-*γ*-lyase inhibitor, propargylglycine (PAG), at 50 mg/kg, i.v. concomitant with the gastric distention. One group of animals received PAG at 50 mg/kg, i.v. + SNP (a NO donor at 6 mg/kg intragastrically) [14] concomitant with the gastric distention. At the end of experiment, animals were killed by cardiac exsanguination. Gastric effluents were collected in chilled tubes, centrifuged at 5000 rpm for 10 min, and kept in −80°C until measurement of the luminal levels of NO. In order to measure mRNA expression of endothelial nitric oxide synthase (eNOS), COX-2, and H^+^/K^+^-ATPase *α*-subunit, the stomachs of animals were removed, opened along the greater curvature, rinsed with physiological saline, and pinned out in ice-cold saline. One hundred milligrams of gastric mucosal tissues was quickly excised, snap-frozen, and stored in liquid nitrogen for mRNA analysis. In the second set of experiments, to show the effect of H_2_S precursor, L-cysteine, on distention-induced gastric acid secretion, 12 rats were randomly divided into L-cysteine-treated and the corresponding control groups (6 in each). They received L-cysteine (50 mg/kg, i.p.) or saline once a day for 5 days before stimulation of the gastric acid secretion. To quantify the mRNA expressions of eNOS, COX-2, and H^+^/K^+^-ATPase *α*-subunit, 100 mg of gastric mucosa was collected, snap-frozen, and stored in liquid nitrogen.

### 2.3. Determining the Basal Acid Secretion and Evaluation of Gastric Acid Secretion

After the surgical preparation, basal gastric acid secretion was allowed to stabilize for at least 30 min. At the end of this period, mean acidity of two first 15 min of gastric effluent considered as basal acid output. The acidity in the gastric washout was measured with an autotitrator pH meter (Radiometer, Copenhagen, Denmark) by automatic potentiometric titration to pH 7 with 0.01 N NaOH and was expressed as *μ*EqH^+^/90 min.

### 2.4. RNA Extraction and cDNA Synthesis

The total RNA was extracted from the frozen tissue samples using TriPure reagent isolation (Roche, Diagnostics). The purity and concentration of the extracted RNA were determined spectrophotometrically at 260 and 280 nm wavelength (Eppendorf, BioPhotometer Plus, Germany). The cDNA was synthesized from one microgram of the total RNA using a cDNA synthesis kit (Bioneer, Daejeon, South Korea) according to the manufacturer's instruction.

### 2.5. Quantitative Real-Time PCR

The mRNA levels of the target (eNOS, COX-2, and H^+^/K^+^-ATPase *α*-subunit) and housekeeping genes, glyceraldehyde-3-phosphate dehydrogenase (GAPDH), were measured by quantitative real-time PCR (qPCR) using step-one systems (Applied Biosystems, USA). The specific primers (Bioneer, Daejeon, South Korea) for measurement of eNOS, COX-2, H^+^/K^+^-ATPase *α*-subunit, and GAPDH were used and the lengths for amplified products were as follows: GAPDH (TGCTGGTGCTGAGTATGTCGTG and CGGAGATGATGACCCTTTTGG, 101 bp), H^+^-K^+^-ATPase *α*-subunit (CCACTAGATCTCTTCTTCAGGAACAGGAT and ACT-ATA-AGC-TTT-TCC-GGA-TCT-CAT-CGT-AG, 129 bp), eNOS (TCCGATTCAACAGTGTCTCCT and ACA-GAA-GTG-CGG-GTA-TGC-TC, 251 bp), and COX-2 (CTCCTCAATACTGGAAAC CTAGCACC and TGGTAGGCTGCGGGTCTTG, 144 bp). All PCR amplifications were performed in duplicate reactions and in final volume of 20 *μ*L containing 2 *μ*L cDNA, 50 nm of specific primers, and 10 *μ*L of Master Mix SYBR Green (2x qPCR Master Mix with SYBR Green I and Rox; Primer design, England) using the following protocol: incubation at 95°C for 10 min to activate DNA Taq polymerase, 40 two-step cycles with 15 s at 95°C for denaturation, and annealing-extension at 60°C for 1 min. In addition, the no-template negative control (H_2_O) was routinely run in every PCR. The melting curve was examined at the end of amplification process to ensure the specificity of PCR products. The purity of each amplicon for each reaction was further confirmed by agarose gel electrophoresis. Expression levels of all eNOS and H^+^/K^+^-ATPase *α*-subunit genes were normalized against GAPDH expression (internal calibrator for equal RNA template loading and normalization). To determine the relative quantification of gene expression, comparative cycle of threshold (Ct) method with arithmetic formulae (2^−ΔΔCt^) was used [16]. Expression in control animals was normalized to 1.

### 2.6. Determining NO Luminal Level

To show the effect of NaHS on NO release, the luminal release of NO from the gastric mucosa was measured indirectly based on the amount of NO metabolites (NO_*x*_), nitrate (NO_2_), and nitrite (NO_3_) using the nitrate/nitrite colorimetric assay kit purchased from Cayman Chemical Company (Ann Arbor, MI, USA). NO_*x*_ levels were measured according to manufacturer's instructions. Briefly, NO_3_ reduced to NO_2_ with nitrate reductase and then nitrite was incubated with Griess reagents for 10 min at room temperature [17]. The absorbance at 540 nm was then measured using a plate reader (Bio-Rad, 680 microplate reader) and the results were expressed as total NO_*x*_ output obtained for 90 min after treatment with NaHS or L-NAME + NaHS.

### 2.7. Statistical Analysis

Data are shown as mean ± S.E.M. Statistical analysis was performed by one-way ANOVA and followed by post hoc Tukey's test. Significance was set at a *P* < 0.05 level.

## 3. Results

### 3.1. Effect of NaHS, L-Cysteine, PAG, and PAG + SNP on Distention-Induced Gastric Acid Secretion

**Figure 1:**
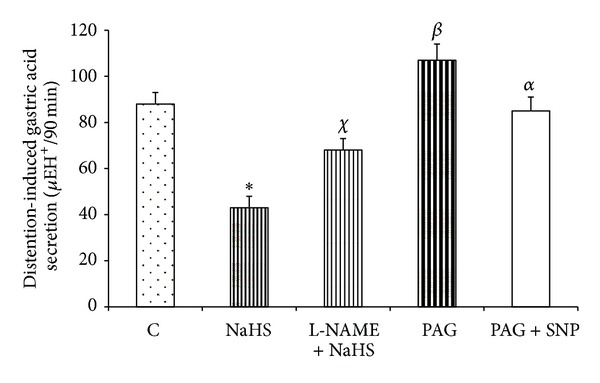
NaHS decreased the gastric acid output induced by distention (1.5 mL/100 g of body weight by normal saline and pH 7 and 37°C for 90 min). C: control, NaHS: animals received NaHS (80 *μ*g/kg, i.v.) just prior to stimulation of the gastric acid secretion, NaHS + L-NAME: animals received L-NAME at 10 mg/kg, i.v., 5 min before the administration of NaHS (80 *μ*g/kg, i.v.), PAG: rats received cystathionine-*γ*-lyase inhibitor, propargylglycine (PAG), at 50 mg/kg, i.v. concomitant with the gastric distention, PAG + SNP: animals received PAG at 50 mg/kg, i.v. + SNP (a NO donor at 6 mg/kg intragastrically) concomitant with the gastric distention. Asterisk indicates a significant decrease (*P < 0.01) as compared to the control group; ^*χ*^ in the above column indicates a significant increase (^**χ**^ *P* < 0.01) as compared to NaHS-treated group; ^**α**^ *P* < 0.05 as compared with PAG + SNP group; ^*β*^ in the above column indicates a significant increase (^*β*^ *P* < 0.01) as compared to the control. Data are expressed as mean ± S.E.M.

**Figure 2:**
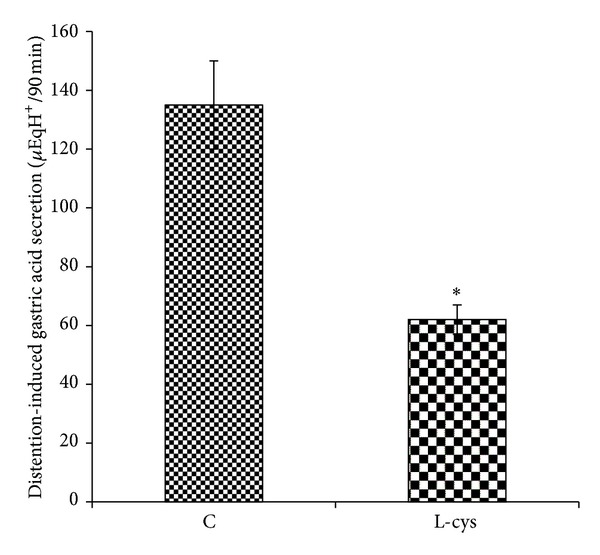
L-Cysteine decreased the gastric acid output induced by distention (1.5 mL/100 g of body weight by normal saline and pH 7 and 37°C for 90 min). Animals received normal saline at 2 mL/kg, i.p. (C: control), or L-cysteine at 50 mg/kg, i.p. (L-cys), once a day for five days before stimulation of the gastric acid secretion. Asterisk indicates a significant decrease (*P < 0.01) as compared to control. Data are expressed as mean ± S.E.M.

As shown in Figure 1, the gastric acid output in response to distention (physiologic saline, 1.5 mL/100 g of body weight) was significantly decreased by an intravenous injection of NaHS (80 *μ*g/kg) or as compared with the control group (*P* < 0.01). It also can be seen from Figure 1 that the inhibitory effect of NaHS on distention-induced gastric acid secretion was significantly decreased (*P* < 0.01) by the pretreatment of L-NAME (10 mg/kg, i.v.). According to Figure 1, the gastric acid output in response to distention significantly increased in PAG-treated rats as compared with the control group (*P* < 0.01). The acid response to distention in PAG + SNP-treated rats was significantly lower than in PAG-treated animals (Figure 1). The acid output in response to distention was also significantly decreased five days before treatment with L-cysteine as compared with the corresponding control group (Figure 2).

### 3.2. Effect of NaHS on Luminal Release of NO

**Figure 3:**
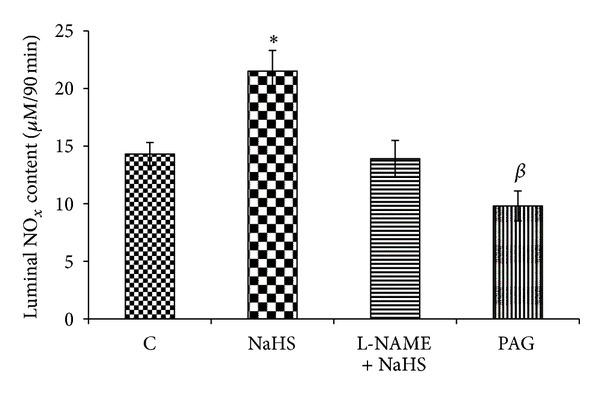
Effect of NaHS treatment on luminal level of nitric oxide. The luminal content of NO was significantly increased by NaHS. C: control, NaHS: animals received NaHS (80 *μ*g/kg, i.v.) just prior to stimulation of the gastric acid secretion, NaHS + L-NAME: animals received L-NAME at 10 mg/kg, i.v., 5 min before the administration of NaHS (80 *μ*g/kg, i.v.), PAG: rats received cystathionine-*γ*-lyase inhibitor, propargylglycine (PAG), at 50 mg/kg, i.v. concomitant with the gastric distention. *P < 0.01 versus the control group; ^*β*^ in the above column indicates a significant decrease (^*β*^ *P* < 0.01) as compared to the control. Data are expressed as mean ± S.E.M.

The luminal level of NO was higher in NaHS-treated animals than in control and L-NAME + NaHS groups. NO luminal level in PAG-treated rats was lower than in NaHS-treated group. As shown in Figure 3, this level was significantly increased in NaHS-treated rats compared with the control rats and PAG-treated animals.

### 3.3. Effect of NaHS and L-Cysteine on mRNA Expressions of H^+^/K^+^-ATPase *α*-Subunit, COX-2, and eNOS

**Figure 4:**
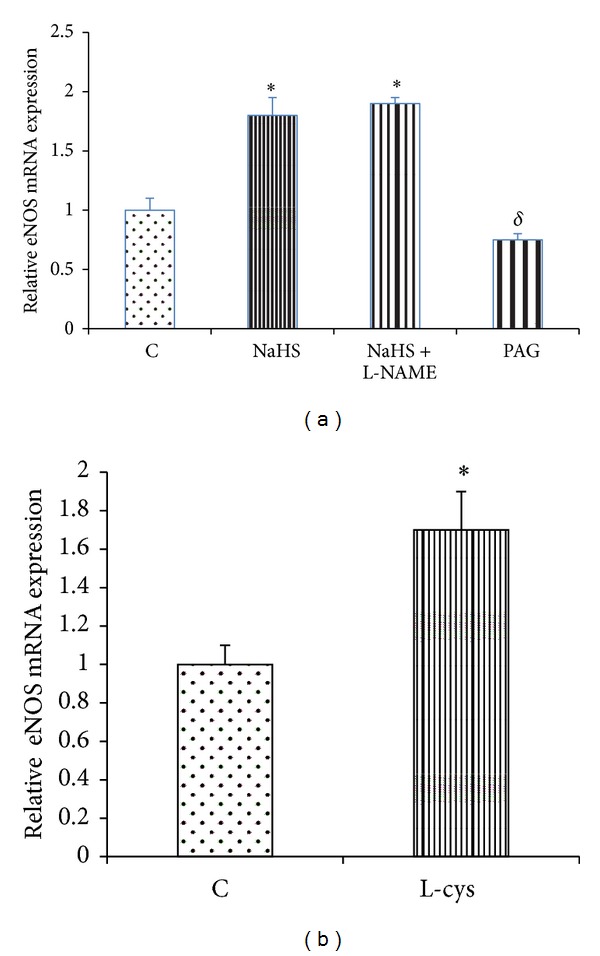
(a) Effect of NaHS on the gastric mucosal mRNA expression of endothelial nitric oxide synthase (eNOS). Analysis of semiquantitative real-time PCR results showed that the administration of NaHS increased the mRNA expression of eNOS. C: control, NaHS: animals received NaHS (80 *μ*g/kg, i.v.) just prior to stimulation of the gastric acid secretion, NaHS + L-NAME: animals received L-NAME at 10 mg/kg, i.v., 5 min before the administration of NaHS (80 *μ*g/kg, i.v.), and PAG: rats received cystathionine-*γ*-lyase inhibitor, propargylglycine (PAG), at 50 mg/kg, i.v. concomitant with the gastric distention. (b) animals received normal saline at 2 mL/kg, i.p. (C: control), or L-cysteine at 50 mg/kg, i.p. (L-cys), once a day for five days before stimulation of the gastric acid secretion. Asterisks indicate a significant increase (*P < 0.01) as compared to the control group;^**δ**^ *P* < 0.05 indicates a significant decrease as compared with the control group. Data are expressed as mean ± S.E.M.

**Figure 5:**
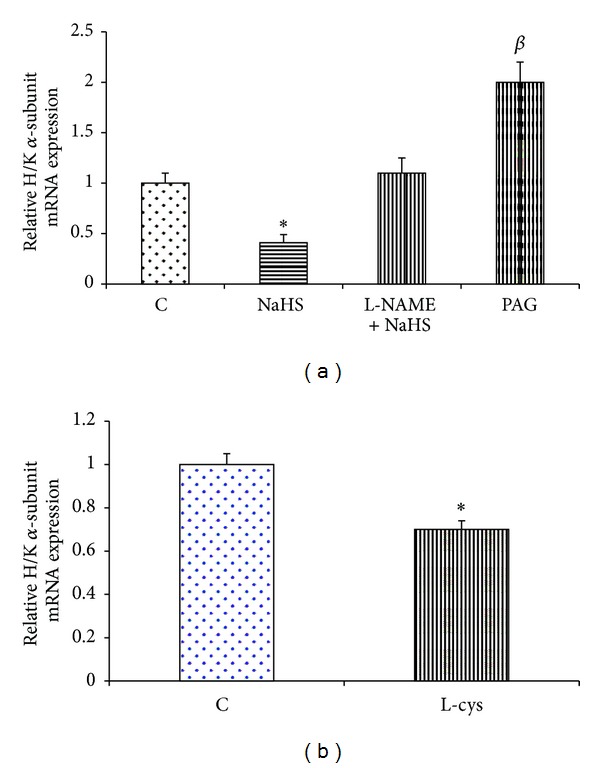
Effect of NaHS (a) and L-cysteine (b) on the gastric mucosal mRNA expression of H^+^/K^+^-ATPase *α*-subunit. (a) Analysis of semiquantitative real-time PCR results showed that the administration of NaHS decreased the mRNA expression of H^+^/K^+^-ATPase *α*-subunit. In contrast, this level increased in PAG-treated rats as compared to the control group. C: control, NaHS: animals received NaHS (80 *μ*g/kg, i.v.) just prior to stimulation of the gastric acid secretion, NaHS + L-NAME: animals received L-NAME at 10 mg/kg, i.v., 5 min before the administration of NaHS (80 *μ*g/kg, i.v.), and PAG: rats received cystathionine-*γ*-lyase inhibitor, propargylglycine (PAG), at 50 mg/kg, i.v. concomitant with the gastric distention. (b) Animals received normal saline at 2 mL/kg, i.p. (C: control), or L-cysteine at 50 mg/kg, i.p. (L-cys), once a day for five days before stimulation of the gastric acid secretion. Asterisks indicate a significant decrease (*P < 0.01) as compared to the control group; ^*β*^ in the above column indicates a significant increase (^*β*^ *P* < 0.01) as compared to the control. Data are expressed as mean ± S.E.M.

**Figure 6:**
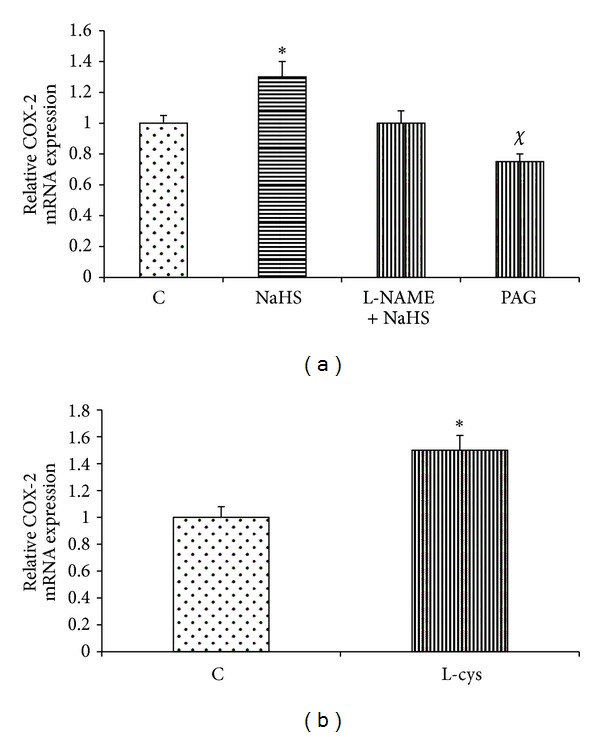
Effect of NaHS (a) and L-cysteine (b) on the gastric mucosal mRNA expression of COX-2. (a) Analysis of semiquantitative real-time PCR results showed that the administration of NaHS increased the mRNA expression of COX-2. C: control, NaHS: animals received NaHS (80 *μ*g/kg, i.v.) just prior to stimulation of the gastric acid secretion, NaHS + L-NAME: animals received L-NAME at 10 mg/kg, i.v., 5 min before the administration of NaHS (80 *μ*g/kg, i.v.), and PAG: rats received cystathionine-*γ*-lyase inhibitor, propargylglycine (PAG), at 50 mg/kg, i.v. concomitant with the gastric distention. (b) Animals received normal saline at 2 mL/kg, i.p. (C: control), or L-cysteine at 50 mg/kg, i.p. (L-cys), once a day for five days before stimulation of the gastric acid secretion. Asterisks indicate a significant increase (*P < 0.01) as compared to the control group; ^*χ*^ *P* < 0.05 indicates a significant decrease as compared with the control. Data are expressed as mean ± S.E.M.

According to Figure 4, the gene expression of eNOS was significantly increased by NaHS and L-cysteine as compared with the corresponding controls. The mRNA expression of eNOS was significantly decreased in PAG-treated rats as compared with the control group (Figure 4). The level of mRNA expression of H^+^/K^+^-ATPase *α*-subunit in control rats was higher than in NaHS- and L-cysteine-treated animals. According to Figure 5, this level was significantly decreased in NaHS- and L-cysteine-treated rats compared with the corresponding control groups. In contrast, the mRNA expression of H^+^/K^+^-ATPase *α*-subunit was significantly increased in PAG-treated rats as compared with the control group (Figure 5). According to Figure 6, the gene expression of COX-2 was significantly increased by NaHS and L-cysteine as compared with the corresponding controls.

## 4. Discussion

It has been shown that hydrogen sulfide increases the release of NO from the vascular endothelium [10]. Ise and coworkers also showed that a mucosal administration of NaHS, a H_2_S donor, increases duodenal release of NO in rats [11]. Nitric oxide has been shown to modulate the acid output in response to gastric distention [14]. The inhibitory effect of NO on gastric acid secretion is mediated by acting directly on the parietal cell or indirectly by inhibiting the release of histamine [13, 18]. Moreover, NO has been reported to increase somatostatin release from rabbit D cells [19], which in turn decreases histamine release from enterochromaffin-like cells (ECL). Nitric oxide has been also demonstrated to inhibit gastric acid secretion in isolated human gastric glands and there is endogenous formation of NO within the glandular epithelium in the vicinity of the parietal cells [13].

As shown in the present study, an intravenous injection of H_2_S donor, NaHS, and five-day pretreatment of H_2_S precursor, L-cysteine, decreased the acid output in response to gastric distention (Figures 1 and 2). The results also showed the antisecretory effect of H_2_S significantly reduced by L-NAME pretreatment. Therefore, the inhibitory effect of H_2_S on gastric acid secretion could partly be mediated through an increase in NO release. To clarify the effect of H_2_S on NO production and release, we first measured the luminal level of NO using a nitrate/nitrite colorimetric assay kit. According to Figure 3, NO content in gastric effluents increases after NaHS treatment. The luminal level of NO in NaHS-treated rats was higher than in control animals and also in L-NAME pretreated rats (Figure 3). This level in PAG-treated rats was significantly lower than in NaHS-treated and control groups. Recently, Ise et al. demonstrated that the stimulatory effect of NaHS on duodenal bicarbonate secretion decreased by L-NAME pretreatment, suggesting the participation of endogenous NO in this action [11]. Therefore, these findings together clearly showed that endogenous and exogenous H_2_S stimulate nitric oxide production/release and show the involvement of nitric oxide in this inhibitory effect of hydrogen sulfide on distention-induced gastric acid secretion. Moreover, the results showed that the acid output in response to distention in PAG + SNP-treated rats was significantly lower than in PAG-treated animals, indicating the involvement of NO in antisecretory effect of H_2_S (Figure 1).

Secondly, to evaluate the effects of endogenous and exogenous H_2_S on eNOS gene expression, the mRNA level of eNOS was measured by semiquantitative real-time PCR. As shown in Figure 4(a), gene expression of eNOS in PAG-treated rats was significantly lower than in control rats. This level in NaHS- and L-cysteine-treated rats was significantly increased as compared with the corresponding control rats (Figures 4(a) and 4(b)). These findings show that endogenous and exogenous H_2_S stimulate eNOS mRNA expression. Thus, the inhibitory effect of H_2_S on gastric acid secretion is partly mediated through an increase in the gene expression of eNOS and at the same time an increase in NO production/release, resulting in the inhibition of acid secretion.

According to Figure 4, eNOS mRNA expression increased in L-NAME + NaHS-treated animals rats as compared with the control rats while the luminal release of NO and also gastric acid output did not change compared with the control group. These results show that NaHS upregulated the gene expression of eNOS like NaHS-treated group but it did not affect the enzyme activity and NO production/release as well as the gastric acid output because of the presence of L-NAME.

According to Figure 1, L-NAME pretreatment could not completely reverse the antisecretory effect of NaHS on distention-induced gastric acid secretion. Therefore, it seems that there is still another factor which is activated by H_2_S and it in turn inhibits or neutralizes the gastric acid. To answer the above mentioned question, we quantified the gene expression of COX-2. Hydrogen sulfide has been shown to upregulate the gene expression of COX-2 and PGE_2_ production in isolated rat cardiomyocytes [20]. Moreover, it has been reported that inhibition of H_2_S production decreases the level of COX-2 expression and PGE_2_ synthesis in the rat colon [21]. It has been shown that the protective activity of H_2_S on ethanol-induced gastric lesions in mice is dependent on the activation of capsaicin-sensitive sensory neurons [22]. Capsaicin has been demonstrated to increase PGE_2_ production and NO release in the duodenum via the activation of these sensory neurons [23, 24]. Consistent with these results, the findings of present study showed that the mRNA expression of COX-2 in gastric mucosal tissue was upregulated in NaHS- and L-cysteine-treated rats as compared with the control (Figure 6) while it significantly decreased in PAG-treated animals. These findings together show that exogenous and endogenous H_2_S increase the prostaglandins production/release. Therefore, due to the antisecretory and acid-neutralizing properties of PGs, it could conclude that a reduction of the acid output/an increase in gastric pH by NaHS in response to distention is partly mediated through an increase in the PGs production.

Sodium nitroprusside, a NO donor, has been shown to decrease the activity of gastric H^+^-K^+^-ATPase in rats [25]. Our findings also showed that an intravenous injection of NaHS and five-day L-cysteine pretreatment significantly decreased mRNA expression of H^+^/K^+^-ATPase *α*-subunit in NaHS-treated rats compared with the corresponding control groups (Figures 5(a) and 5(b)). According to Figure 5, the inhibitory effect of NaHS on mRNA expression of H^+^/K^+^-ATPase was abolished by L-NAME pretreatment, suggesting the involvement of NO. Therefore, these results suggest that the other possible antisecretory mechanism of NaHS on gastric acid secretion might be mediated by reducing mRNA expression of H^+^/K^+^-ATPase *α*-subunit.

What is the clinical implication? Recently, there is a growing body of text that has been demonstrated by H_2_S that acts as a rescue molecule for mucosal defense. Till now the mechanisms underlying the gastroprotective effects of hydrogen sulfide were attributed to maintenance and/or elevation of gastric mucosal blood flow [7], stimulation of bicarbonate secretion [11], reduction of proinflammatory cytokine expression/release [9, 26], increase of prostaglandin synthesis [19], decrease of reactive oxygen metabolite production [21], and enhancement of tissue repair [8, 27]. The findings of the present study showed that the antisecretory effect of H_2_S could be the other possible protective mechanism.

What is the physiologic role of endogenous H_2_S in regulation of gastric acid secretion? The enzyme activity and mRNA expression of CSE have been shown in the rat stomach [28]. Recently, we have shown that pharmacological inhibition of CSE by preadministration of PAG leads to an increase in the gastric acid output in response to distention compared to corresponding control [15]. As shown in Figure 1, the results of present study were also confirmed by the previous findings that suggest that the CSE activity and H_2_S production increase concomitant of gastric acid stimulation and decrease the acid output in response to gastric distention. According to Figure 5, mRNA expression of H^+^/K^+^-ATPase *α*-subunit in PAG-treated rats significantly increased as compared to control group. The results together suggest that, concomitant with stimulation of the gastric acid secretion, the CSE activity and H_2_S production/release increase and subsequently decrease mRNA expression of H^+^/K^+^-ATPase *α*-subunit (Figures 1 and 5). These results suggest a housekeeping role for H_2_S to maintain the gastric mucosal integrity by inducing bicarbonate secretion as shown by the previous studies [11] and at the same time by reducing the gastric acid output as shown by the current study.

Moreover, the endogenous H_2_S has been shown to excite the contraction of gastric smooth muscles in mouse and guinea-pig [29, 30]. Therefore, the results of the present study along with the previous reports about the effect of H_2_S on gastric mucosal defense as well as on gastric motility provide new concepts from the physiologic role of H_2_S in the stomach.

The results of the present study clearly showed that an increase in NO concentration and prostaglandins release resulted from the administration of NaHS inhibited and neutralize the gastric acid output. The findings also suggest that H_2_S exerts both an antisecretory effect by reducing the acid output as shown by the present work and at the same time an antacid activity by inducing bicarbonate production as shown by the previous reports [11].

In conclusion the present study showed the following. (i) Administration of NaHS, a H_2_S donor, and L-cysteine, H_2_S precursor, reduced the acid output in response to gastric distention. (ii) Pretreatment with L-NAME mitigated the inhibitory effect of NaHS on distention-induced gastric acid secretion. (iii) The mRNA level of eNOS and COX-2 was significantly increased by NaHS and L-cysteine. (iv) The gene expression of H^+^/K^+^-ATPase *α*-subunit was significantly decreased by NaHS and L-cysteine. (v) The luminal level of nitric oxide was increased by NaHS.
